# Recent Updates on Electrogenerated Hypervalent Iodine Derivatives and Their Applications as Mediators in Organic Electrosynthesis

**DOI:** 10.3389/fchem.2022.883474

**Published:** 2022-04-13

**Authors:** Chaoyue Chen, Xin Wang, Tinghai Yang

**Affiliations:** ^1^ School of Chemistry and Environmental Engineering, Jiangsu University of Technology, Changzhou, China; ^2^ State Key Laboratory of Coordination Chemistry, School of Chemistry and Chemical Engineering, Nanjing University, Nanjing, China

**Keywords:** hypervalent iodine reagent, organic electrosynthesis, anodic oxidation, redox mediator, synthetic method

## Abstract

With the renaissance of chemical electrosynthesis in the last decade, the electrochemistry of hypervalent iodine compounds has picked up the pace and achieved significant improvements. By employing traceless electrons instead of stoichiometric oxidants as the alternative clean “reagents”, many hypervalent iodine compounds were efficiently electro-synthesized *via* anodic oxidation methods and utilized as powerful redox mediators triggering valuable oxidative coupling reactions in a more sustainable way. This minireview gives an up-to-date overview of the recent advances during the past 3 years, encompassing enhanced electrosynthesis technologies, novel synthetic applications, and ideas for improving reaction sustainability.

## Introduction

Hypervalent iodine reagents (HIRs) have recently attracted considerable attention in organic synthesis because of their valuable oxidizing abilities, superior leaving group abilities, unique electrophilic properties, and environment-friendly features (Yoshimura and Zhdankin, 2016; Wang and Studer, 2017). To date, their synthetic applications have expanded from ligand transfer reactions, oxidative coupling processes, halogenations, oxidative rearrangements, heterocycle synthesis, and numerous other reactions (Chen et al., 2020; Takenaga et al., 2020). Furthermore, the applications of chiral HIRs and asymmetric precursors have resulted in many valuable contributions to modern asymmetric synthesis (Yang et al., 2021).

HIRs are often considered as environmentally benign alternatives to various chemical oxidants, especially the highly toxic heavy-metal oxidizers, i.e., Pb(IV), Hg(II), and Tl(III) reagents (Yoshimura and Zhdankin, 2016; Takenaga et al., 2020). Nevertheless, the current common preparation methods of HIRs pose environmental concerns to their widespread application and upscaling (Liu et al., 2018; Elsherbini and Wirth, 2018; Frey et al., 2022). Typically, these preparation methods often require (over)stoichiometric amounts of hazardous or costly oxidants, such as *m*-chloroperbenzoic acid (*m*-CPBA), CH_3_CO_3_H, NaBO_3_, NaIO_4_, oxone (2KHSO_5_·KHSO_4_·K_2_SO_4_), H_2_O_2_, and Selectfluor, thus inevitably leading to an excess of waste products (Francke et al., 2018; Francke, 2019; Elsherbini and Wirth, 2018). Hence, more straightforward and sustainable methods for HIRs preparation and derivatization are highly desired.

Nowadays, organic electrosynthesis has witnessed a considerable renaissance for the excellent redox efficacy and tunable cell conditions (Francke and Little, 2014; Yan et al., 2017; Moeller, 2018). Because this methodology utilizes electrons instead of stoichiometric oxidants/reductants as alternative clean redox reagents and can produce important intermediates or reagents *in situ*, so, organic electrosynthesis is recognized as a reliable and sustainable alternative to conventional approaches (Pollok and Waldvogel, 2020; Novaes et al., 2021).

With the renaissance of organic electrosynthesis, many synthetic chemists have switched to the electrochemical preparation of HIRs *via* the anodic oxidation of iodine (I) precursors, and attempted to use *in situ* electrogenerated HIRs as important mediators for various iodine (III)-mediated transformations (Francke et al., 2018; Elsherbini and Wirth, 2018; Francke, 2019; Francke, 2021). And this research front has undergone a significant expansion and currently become an increasingly active research area of hypervalent iodine chemistry. Given the importance and relevance envisaged in future transformations, herein, we intend to summarize recent applications of electrogenerated HIRs and their applications as redox mediators in various chemical transformations. Due to the limited space, this minireview will only highlight the advances over the past 3 years and serves as an update to the earlier reviews conducted by Francke et al. (Francke et al., 2018; Francke, 2019) and Elsherbini and Wirth (2018), respectively.

## Electrochemical Synthesis of Hypervalent Iodine Reagents

### Electrochemical Synthesis of Hypervalent Iodine (III) Compounds

The first example of the electrochemical synthesis of hypervalent iodine derivatives can be traced back to 1960 by the work of Schmidt and Meinert (1960). In a brief report, they described a procedure for electrochemical generation of well-known (difluoroiodo)benzene (PhIF_2_) by anodic oxidation of iodobenzene in the presence of silver fluoride. Shortly after that, the first electrochemical preparation of diaryliodonium salts was disclosed by Miller and Hoffmann in 1967 (Miller and Hoffmann, 1967). (Dialkoxyiodo)arenes, another traditional iodine (III) reagents, were first electro-synthesized by Nishiyama and Amano in 2006 (Amano and Nishiyama, 2006). Apart from these pioneering researches, only a few scattered reports on electro-generation of aryl iodide(III) species are available.

**SCHEME 1:**
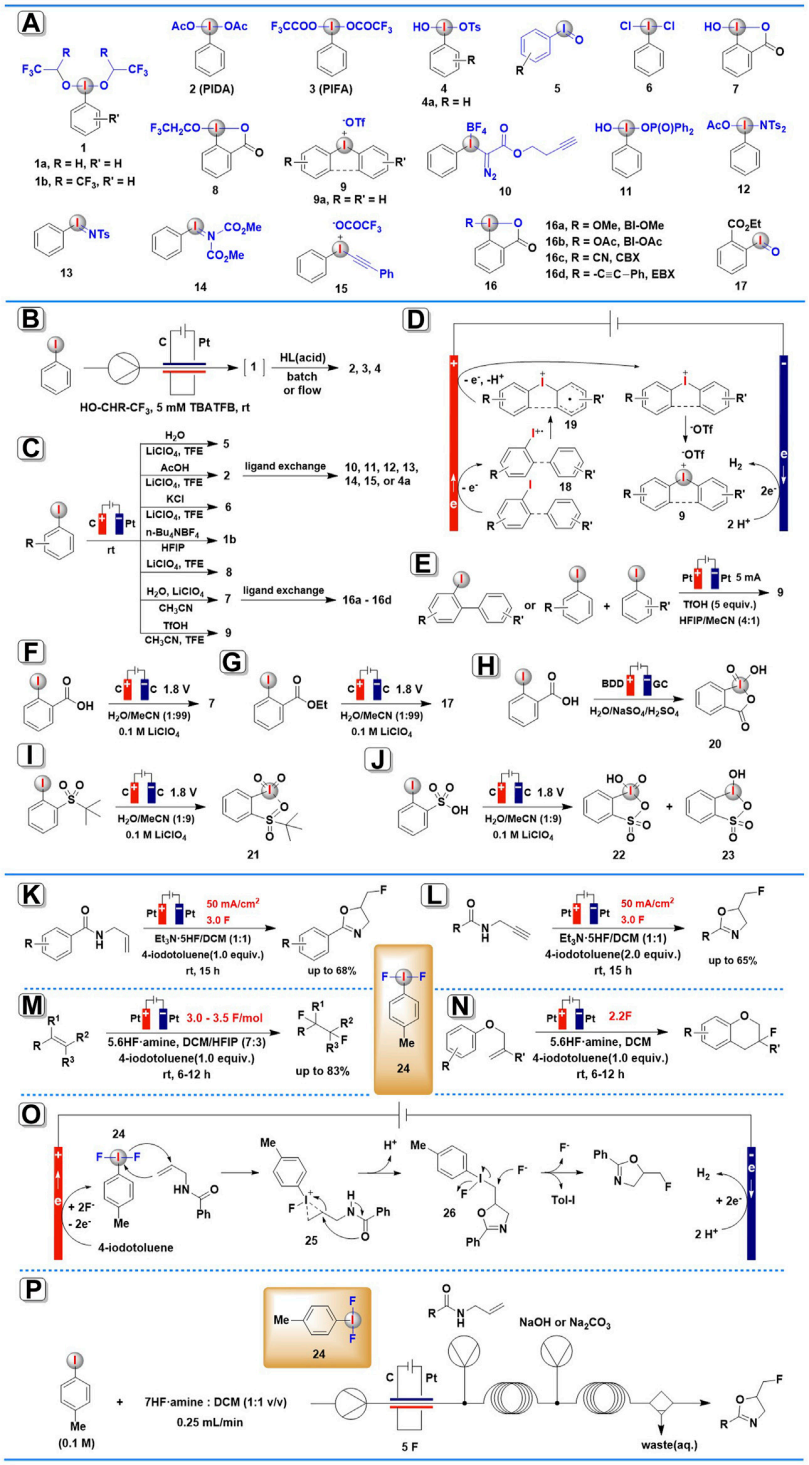
Electrochemical synthesis of HIRs and organic transformations mediated by anodically generated (difluoroiodo)arene. **(A)** Electrogenerated hypervalent iodine derivatives. **(B)** The continuous-flow electrochemical generation of HIRs. **(C)** Anodic oxidation for the synthesis of aryliodine(III) reagents. **(D)** The possible mechanism for electrochemical synthesis of diaryliodonium salts. **(E)** Scalable electrochemical synthesis of diaryliodonium salts. **(F,G)** Electrochemical synthesis of HIRs using water as the O-atom source. **(H–J)** Electrochemical synthesis of organic iodine (V) compounds. **(K–N)** Organic transformations mediated by anodically generated (difluoroiodo)arene. **(O)** The possible mechanism for electrochemical fluorocyclization of N-allylcarboxamides mediated by ArIF_2_. **(P)** Fluorinations using flow electrolysis.

During the past decade, with the development of equipment and technologies in electrochemistry, the efforts on electrochemical synthesis of hypervalent iodine reagents have picked up the pace and achieved significant improvements (Scheme 1).

In 2019, the first generic method for synthesizing bench-stable classic HIRs *via* anodic oxidation-ligand exchange in an electrochemical flow reactor was described by Wirth and co-workers (Elsherbini et al., 2019). By using a glassy carbon anode and a platinum cathode, iodoarene precursors were anodically oxidized to the corresponding active (dialkoxyiodo)arenes **1** (Scheme 1A) either in 1,1,1,3,3,3-hexafluoro-2-propanol (HFIP) or in 2,2,2-trifluoroethanol (TFE) under continuous-flow conditions (Scheme 1B). Nevertheless, the electrogenerated (dialkoxyiodo)arenes **1** are only stable in solution, and they disintegrate as soon as the solvent is removed, so, *in situ* electrochemically generated **1** commonly needs to be immediately used in the next transformation. When unstable **1a** or **1b** been further treated with the appropriate acids, many useful stable HIRs, such as (diacetoxyiodo)benzene (PIDA, **2**), [(bistrifluoroacetoxy)iodo]benzene (PIFA, **3**), and Koser’s reagent and its derivatives **4** were achieved *via* facile ligand exchange reactions. Furthermore, this protocol allowed for the simple synthesis of electron-deficient HIRs that were previously difficult to prepare using conventional methods. In addition, the productivity of this procedure (electrochemical oxidation-ligand exchange) is higher than the similar transformations under batch electrolysis conditions.

Very recently (2021), He and others (Zu et al., 2021) have presented a comprehensive investigation on the usage of anodic oxidation of aryl iodides in the synthesis of diverse hypervalent iodine compounds (Scheme 1C). They provided a sustainable and broadly applicable approach for accessing four types of aryl iodide(III) reagents: iodosylarenes, (difunctionaliodo)arenes, benziodoxoles, and diaryliodonium salts, among others. After a careful examination of the anodic oxidation conditions, electrochemical synthesis of iodosylarenes **5** was carried out in an undivided cell equipped with a graphite anode and a Pt plate cathode and using LiClO_4_ as the electrolyte and TFE as the solvent under a constant current of 15 mA. Three traditional (difunctionaliodo)arenes, PIDA **2**, IBD (PhICl_2_, **6**), and PhI[OCH(CF_3_)_2_]_2_ **1b**, could also be produced in good to outstanding yields with great selectivity by simply substituting H_2_O with AcOH, KCl, or replacing the solvent to HFIP in the absence of H_2_O. 2-Iodobenzoic acids precursors could be efficiently converted into benziodoxoles **7** and **8** in high yields under slightly modified electrochemical conditions. Furthermore, a variety of diaryliodonium salts **9** were obtained in satisfying yields employing TfOH as both the electrolyte and counteranion, and CH_3_CN and TFE as the mixed solvent. When subjected to additional ligand-exchange, the electrogenerated PIDA **2** or hydroxy benziodoxolone **7** could readily convert into many useful HIRs, including Koser’s reagent **4**, Weiss’ reagent **10**, [hydroxy(phosphoryloxy)iodo]benzene **11**, amidoiodane **12**, iodonium imide **13**, alkynyliodonium salt **14**, aryl iodonium ylide **15**, and benziodoxole reagents **16** (BI-OAc, BI-OMe, CBX, and EBX).

The authors suggested two mechanisms to explain the electro-generation of HIRs, one for three types of iodosylarenes, (difunctionaliodo)arenes, and benziodoxoles, and the other for diaryliodonium salts. In the first case, the aryl iodide(III) reagents are proposed to proceed *via* two-electron anodic oxidation of iodoarene in the presence of a suitable ligand. The cathodic half-reaction is the reduction of protons, which produces H_2_ as a by-product. In the latter case (Scheme 1D), one electron anodic oxidation of iodoarene(I) affords radical cation intermediate **18**, which is then converted into intermediate **19** by radical addition with arene species. Subsequently, the resulting intermediate **19** performs a second one-electron anodic oxidation and further combines with ^–^OTf ion to yield the desired diaryliodonium salts **9**.

Almost at the same time, Moran and co-workers also established an electrochemical approach to synthesis cyclic and acyclic diaryliodonium salts **9** (Elsherbini and Moran, 2021). In a simple undivided electrolysis cell in MeCN-HFIP-TfOH without any added electrolyte salts, more than 30 diaryliodonium salts **9** were achieved in high to excellent yields *via* anodic oxidation of iodobiaryls and iodoarene/arene (Scheme 1E). It was worth noting that this protocol has high scalability, in less than 3 h, the reaction was scaled up to a 10 mmol scale, yielding more than 4 g of **9a** (>95%). The large-scale experiment’s solvent mixture was easily recovered (>97%) and recycled several times without a notable drop in yield.

Also in 2021, Folkman and others have demonstrated a novel electrochemical synthetic route to HIRs using water as an abundant, green chemistry O-atom source (Folkman et al., 2021). The bulk electrolysis was conducted in a U-cell equipped with a glassy carbon electrode at 1.8 V vs. Ag/AgNO_3_, using 0.1 M LiClO_4_ as the supporting electrolyte and 1% H_2_O in MeCN as the O-atom source (Schemes 1F,G). IBA (**7**) and its derivative (IBA-ester, **17**) were obtained with Faradaic efficiency of 89 and 78%, respectively. According to isotopic (^18^O) labeling studies using H_2_^18^O, the authors suggest that the O-atom in the hypervalent product is initially coming from the oxidized, O-atom-containing surface of the glassy carbon electrode, and then the surface oxide of the glassy carbon is regenerated by the O-atoms from water solution. These results provide evidence that H_2_O serves as the oxygen source for the newly formed functional groups in hypervalent iodine products in this electrosynthesis process.

### Electrochemical Synthesis of Hypervalent Iodine (V) Compounds

Whereas several methods for electrochemical synthesis of iodine (III) compounds have been developed during the past few decades, the methods for anodic electrosynthesis of iodine (V) species were less explored and have just recently been established.

In 2018 Bystron’s group reported the first electrochemical synthesis of organic iodine (V) compound, 2-iodoxybenzoic acid (IBX, **20**), through anodic oxidation of 2-iodobenzoic acid (IBA) in a divided cell using a boron-doped diamond (BDD) electrode in 0.2 M H_2_SO_4_ aqueous solution (Bystron et al., 2018; Devadas et al., 2020) (Scheme 1H). This anodic oxidation process exhibited excellent selectivity and high current efficiency (>50%). No undesired organic products were generated with O_2_ as the only by-product. The aqueous environment was recognized as a crucial aspect of this anodic oxidation process because iodine (V) compounds could be electrochemically generated only in aqueous electrolyte solutions (Devadas et al., 2020).

Apart from iodine (III) compounds, the aforementioned method by Folkman and others (Folkman et al., 2021) also enables the electrochemical synthesis of iodine (V) species (Schemes 1I,J). In the same paper, the widely used IBX-sulfone **21** and a mixture of IBA-sulfonic acid **22** with IBX-sulfonic acid **23** were obtained *via* bulk anodic oxidation electrolysis of PhI-sulfone and PhI-sulfonic acid using water as the O-atom source. However, attempts to isolate these iodine (V) derivatives were unsuccessful due to decomposition under experimental conditions.

## Hypervalent Iodine Derivatives as Redox Mediators in Organic Electrosynthesis

**SCHEME 2:**
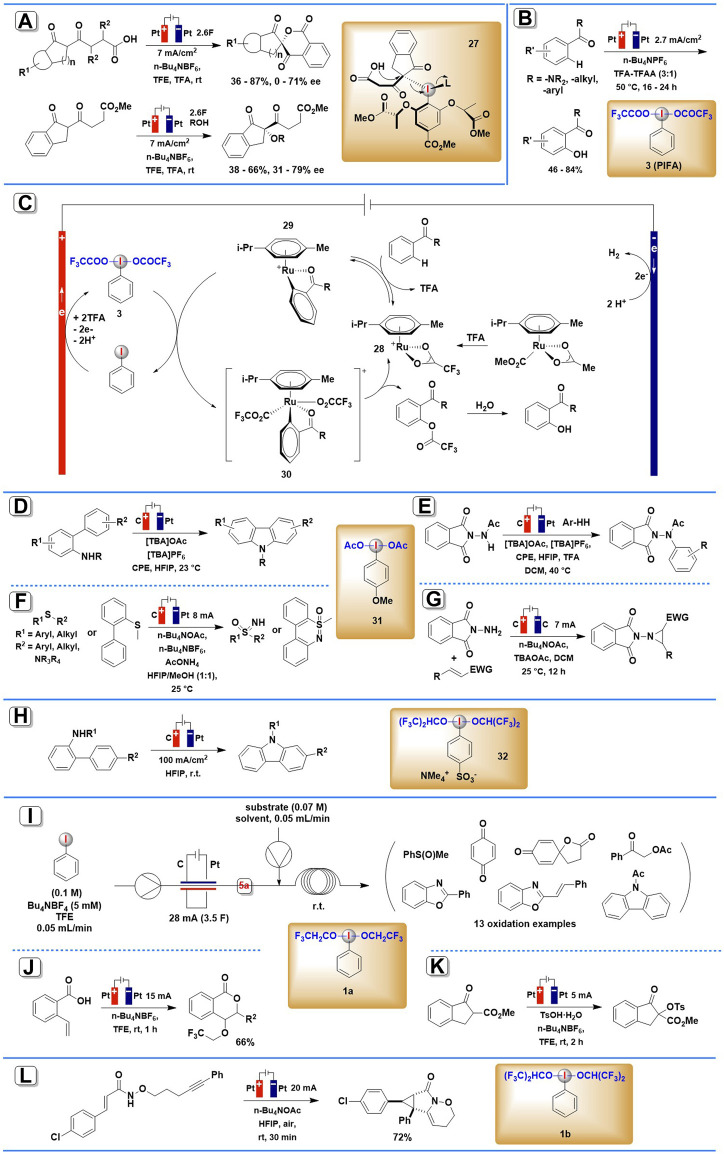
Organic transformations mediated by anodically generated (diacyloxyiodo)arenes or (dialkoxyiodo)arenes. **(A)** The asymmetric spirolactonization mediated by anodically generated chiral iodine (III) intermediate. **(B)** The reaction and the possible mechanism **(C)** of iodine(III)/Ruthenium complexes electrocatalyzed C–H oxygenation. **(D,E)** The electrocatalytic C-N coupling mediated by anodically generated ArI(RCOO)_2_. **(F)** The electrochemical synthesis of NH-sulfoximines and NH-sulfonimidamides mediated by anodically generated ArI(RCOO)_2_. **(G)** The electrochemical aziridination mediated by anodically generated ArI(RCOO)_2_. **(H)** The intramolecular oxidative C-N bond formation mediated by anodically generated (dialkoxyiodo)arene. **(I–K)**Organic transformations mediated by anodically generated PhI(OCH_2_CF_3_)_2_. **(L)** The electrochemical synthesis of cyclopropane mediated by anodically generated (dialkoxyiodo)arene.

In many organic electrosynthesis practices, HIRs which *in situ* generated at the anode, often immediately used as in-cell or ex-cell or in-flow mediators in subsequent redox reactions, thus efficiently triggering many valuable organic transformations, including fluorination, oxidative cyclization, trifluoroethoxy-lactonization, aziridination, and enantioselective cyclizations, etc. (Schemes 1K–P, 2). Since these reactions are not suitable to be classified according to the type of reaction or the type of chemical bond formed, they are classified and described herein according to the iodine (III) redox mediators employed.

### Electrochemically Generated (Difluoroiodo)arene ArIF_2_ as Redox Mediators

A novel electrochemical fluorocyclization of N-allylcarboxamides for the construction of 5-fluoromethyl-2-oxazolines was developed in 2019 by Waldvogel and co-workers (Haupt et al., 2019). The reaction was carried out under constant current in an undivided cell charged with two Pt electrodes and anodically generated ArIF_2_ **24** was employed as the redox mediator in the presence of Et_3_N·5HF (Scheme 1K). It was noteworthy that the iodoarene precursors could be partly or even quantitatively isolated after electrolysis.

The proposed mechanism (Scheme 1O) showed that 4-iodotoluene is oxidated at the anode to generate the activated ArIF_2_ **24**, which is then transformed to a cyclic iodonium species **25** by addition with an allyl moiety. The intermediate formed undergoes intramolecular ring-opening leading to 5-(λ^3^-iodanyl)methyl oxazoline **26**, which then suffers an *SN*2 nucleophilic attack from fluorine, resulting in the formation of the halogenated product along with the regeneration of ArI.

Encouraged by these findings, the same group continued to explore more organic transformations mediated by electrochemically generated ArIF_2_ **24**, a similar electrochemical protocol (Scheme 1L) has been successful in the conversion of a wide range of N-propargylamides into their corresponding 5-fluoromethyl-2-oxazoles (Herszman et al., 2019). It was noted by the authors that the stoichiometry of iodobenzene ArI proved to be a crucial factor in determining product selectivity. In addition, this electrochemical approach allows the fluorocyclization of electron-rich N-propargylamides that are difficult or impossible in conventional reagent-based methods. The proposed mechanism showed that the fluorocyclization proceeds through ArIF_2_ **24**, which is generated by direct electrochemical oxidation of iodoarene at the anode.

In 2020, Lennox and co-workers report a method for electrochemical vicinal 1,2-difluorination of alkenes using Tol-IF_2_ **24** as a mediator (Doobary et al., 2020). In this case, fluorinated products yielded a range from moderate to outstanding across a wide range of alkene substrates (Scheme 1M). Superior to conventional methods, this ex-cell approach permits the difluorination of electron-rich moieties, anilines, and substituted internal alkenes. The authors noted that the electrochemical synthesis of a hypervalent iodine mediator *via* an ex-cell approach, which avoids oxidative substrate degradation, is the key factor for success.

Based on this result, Lennox’s group continued to utilize this electrochemical approach for intramolecular fluoroarylation of phenolic ethers mediated by electrochemically generated ArIF_2_ **24** (Doobary et al., 2021) (Scheme 1N). The use of an ex-cell procedure has proven to be critical to the success of this process. Notably, this electrochemical approach exhibited broad functional group compatibility, even electron-withdrawing groups on the aromatic ring and gem substituents on the alkene were well tolerated in the reaction system. Furthermore, this reaction could be easily scaled up with good efficiency, which is important for future applications.

Unlike the batch approaches outlined above, Wirth and co-workers described a continuous flow method for electrochemical generation of ArIF_2_ **24** and its synthetic applications in 2021 (Winterson et al., 2021). Several fluorination transformations, including fluorocyclizations, difluorinations, monofluorinations, iodofluorinations, and ring contractions, are conveniently implemented in continuous flow (Scheme 1P). Because (difluoroiodo)arene mediators are not bench-stable and degrade as soon as the solvents are removed, their continuous generation and immediate application in flow are particularly useful. Under high flow rates, the continuous-flow procedure achieved higher yields and up to 834 mg h^−1^ productivities with drastically decreased reaction times.

### Electrochemically Generated (Diacyloxyiodo)arene ArI(RCOO)_2_ as Redox Mediators

The first application of electrogenerated chiral hypervalent iodine compound in enantioselective electrosynthesis (Scheme 2A) was developed by Wirth and co-workers in 2019 (Gao et al., 2019). The asymmetric spirolactonization process utilized an undivided cell under constant current reaction conditions (*J* = 7 mA/cm^2^) with platinum electrodes and the addition of 1.2 equiv. of mediator and Bu_4_NBF_4_ electrolyte. Fluorinated alcohol TFE was chosen as the solvent for stabilizing iodine (III) reagents generated by the anodic oxidation of iodoarenes. Under these conditions, a range of diketo carboxylic acids were smoothly converted into chiral six-membered lactones in 36–87% yield and 0–71% *ee* mediated by *in situ* generated chiral iodine (III) intermediate **27**. Intermolecular *α*-alkoxylation of diketo ester derivatives can also be achieved using the same protocol, and the corresponding coupling products were obtained in 38–66% yield and 31–79% *ee*. Furthermore, this batch-type process could be adapted to an electrochemical flow microreactor to reduce or minimize the use of supporting electrolytes.

In 2020, Ackermann and co-workers demonstrated how iodoarene mediators can be integrated into metallic-electrocatalytic processes (Massignan et al., 2020). By a combination of the catalytic electro-regeneration of iodine (III) intermediate **3** with ruthenium-(II) catalyzed C-H activation, a wide array of aromatic amides and ketones were selectively converted to their corresponding oxygenated products (Scheme 2B). The reaction was carried out in an undivided cell equipped with Pt plate electrodes under a relatively low current density (2.7 mA/cm^2^), using a mixture consisting of TFA, TFAA, and n-Bu_4_PF_6_ served as electrolyte. Chemical oxidants such as *m*-CPBA or ozone, which are commonly employed to synthesize the HIRs, failed to give the desired products in adequate yields. Mechanistic studies by experiment, computation, cyclic voltammetry, and in operando NMR spectroscopy showed that an *in situ* formed HIR served the dual role of electron-shuttle and a transfer reagent of carboxylate anions. The proposed mechanism is shown in Scheme 2C. The catalytic cycle starts with ruthenium-(II)-catalyzed C-H activation of **28**, and then the resulting complex **29** suffer indirect oxidation by anodically generated PIFA, leading to the formation of ruthenium(IV) intermediate **30**. The ruthenium(IV) intermediate undergoes reductive elimination, releasing the product and closing the catalytic cycle.

Also in 2020, Powers and others described electrochemical C–N coupling methodology to achieve N-acetylcarbazole mediated by anodically generated iodine (III) intermediate **31** (Maity et al., 2020). An undivided cell was equipped with a glassy carbon electrode at the anode and a platinum electrode at the cathode using HFIP as solvent at room temperature (Schemes 2D,E). This electrosynthesis process can apply to both intra- and inter-molecular C–H/N–H coupling. The crucial significance of acetate ions in stabilizing initially produced iodanyl radical intermediates has been demonstrated in mechanistic investigations.

An efficient synthesis of NH-sulfoximines, NH-sulfonimidamides, and dibenzothiazines *via* anodically generated iodine (III) intermediate **31** was reported by Wang, Xu, and co-workers in 2021 (Kong et al., 2021). The reaction was conducted in an undivided cell equipped with graphite (C) anode and Pt plate cathode, at a constant current of 8 mA using *n*-Bu_4_NPF_6_ as the electrolyte (Scheme 2F). It is noteworthy that this in-cell method employs catalytic amounts of iodoarene and simple ammonia sources, thus avoiding the use of an excess of HIRs.

In 2021, Dai, Xu, and co-workers reported a protocol for electrochemical aziridination of electron-deficient alkenes mediated by electro-generated iodine (III) species **31** (Liu et al., 2021). By using graphene sheet as an anode, graphite felt as cathode, and n-Bu_4_NBF_4_ as supporting electrolyte, a number of aziridines were smoothly achieved in DCM under a current of 7 mA (Scheme 2G). A broad substrate scope was achieved in this protocol, including various cinnamate, cinnamide, *α*,*β*-unsaturated nitrile, and ketones.

### Electrochemically Generated (Dialkoxyiodo)arene as Redox Mediators

In 2019, Francke’s group developed an electrochemical protocol to achieve intramolecular oxidative C–N bond formation (Roesel et al., 2019). In this protocol, a novel ionically tagged iodophenylsulfonate was employed as both mediator and supporting electrolyte, thus allowing electrolysis without supporting electrolyte additives and facilitating the recovery from the reaction mixture (Scheme 2H). Anodic oxidation of 4-iodobenzenesulfonate led to the formation of redox-active iodine (III) species **32**, which could trigger the oxidative cyclization of 2-acetamidobiphenyls.

Wirth and co-workers demonstrated that the electrochemical generator of iodine (III) **1a** exhibits excellent efficiency and reliability in organic electrosynthesis (Elsherbini et al., 2019). By coupling the flow of the *in situ* electrochemically generated PhI(OCH_2_CF_3_)_2_ with the second flow of thioanisoles, hydroquinone, 3-(4-hydroxyphenyl)propanoic acid, *ortho*-hydroxyimines, acetophenones, propiophenones, or acetamide in TFE solvent, a series of oxidative transformations including mono-oxidation, dearomatization, cyclization, *α*-acetoxylation, tosyloxylation, etc. were successfully achieved using simple reaction setup (Scheme 2I). The yields of the end products are normally high, and they can be easily enhanced by adjusting the second reaction’s residence time and temperature. Furthermore, all these reactions can be scaled up to 0.4–1.0 mmol.

Based on their method for the electrochemical synthesis of aryl iodide(III) reagents mentioned above, He and his co-workers further developed two oxidative transformations, trifluoro-ethoxylactonization, and tosyloxylation, enabled by anodically generated iodine (III) intermediate **1a** (Zu et al., 2021). The two reactions were both conducted in an undivided cell equipped with platinum electrodes under a constant current of 5 mA, using n-Bu_4_NBF_4_ as the electrolyte and TFE as the solvent (Schemes 2J,K). The corresponding products were achieved in moderate yields.

In 2022, Wright, Wen, Zhang, and co-workers reported one example of cation-induced [2 + 1] cyclization of alkenyl amide mediated by electrochemically generated iodine (III) intermediate (Yuan et al., 2022). The iodine (III) species **1b**, which *in situ* generated from iodobenzene in the presentence of n-Bu_4_NOAc in HFIP, smoothly promoted the annulation process and constructed highly strained cyclopropane-fused lactam in 72% yield (Scheme 2L).

## Summary and Outlook

As shown herein, the growing interest in hypervalent iodine chemistry together with the “renaissance” of organic electrosynthesis opened new possibilities for developing synthetically attractive transformations in a more sustainable way. By means of anodic oxidation of iodine (I) precursors, HIRs can be electrochemically generated using electric current as an inexpensive and traceless oxidant under environmentally benign conditions. The *in situ* generated HIRs can utilize as powerful batch or flow mediators and trigger valuable oxidative coupling reactions, including fluorocyclizations, trifluoro-ethoxylactonisation, aziridination, asymmetric spirolactonization, etc.

Despite considerable progress and achievements, there are major challenges still yet to be overcome. Firstly, the number and types of electrochemically accessible HIRs, especially iodine (V) species, still need to be expanded. Moreover, the development of low-cost, non-toxic, and more sustainable electrolytes as a replacement of commonly used NEt_3_·nHF electrolytes or fluorinated alcohols is highly desirable. Finally, the exploration of more challenging electro-synthetic applications, especially asymmetric electrocatalysis, mediated by electro-generated iodine (III) or iodine (V) is also in urgent demand. Although electrochemistry of hypervalent iodine compounds is still in its infancy, the authors believe that this research front may enjoy imminent prosperity in the near future.
